# Effect of tonsillectomy in a child with obesity and obstructive sleep apnea: A case report and review of the literature

**DOI:** 10.3389/fped.2022.1101267

**Published:** 2023-01-25

**Authors:** Paola Di Filippo, Greta Orlandi, Giampiero Neri, Sabrina Di Pillo, Francesco Chiarelli, Nadia Rossi, Marina Attanasi

**Affiliations:** ^1^ Department of Pediatrics, University of Chieti, Chieti, Italy; ^2^ Department of Otorhinolaryngology, University of Chieti, Chieti, Italy

**Keywords:** OSA (obstructive sleep apnea), obesity, sleep disturbance, sleep-disordered breathing (obstructive/central sleep apnea), adenotonsilar hypertrophy, tonsillectomy

## Abstract

Obstructive sleep apnea (OSA) is an increasingly recognized disorder in children. Adenotonsillectomy is the primary surgical treatment for OSA in children with adenotonsillar hypertrophy (ATH). We present the case of an obese 4-year-old boy hospitalized for severe desaturation during sleep and severe ATH. Nasal steroid therapy proved ineffective with persistent symptoms. Polygraphy documented severe OSA with an apnea–hypopnea index (AHI) equal to 11. Tonsillectomy resulted in prompt symptom improvement and a substantial reduction of the AHI (2.2). In this case, tonsillectomy alone resulted effective in treating OSA, despite obesity. We concluded that the presence of obesity should not postpone/exclude surgical treatment of preschool children for whom ATH is the most important cause of OSA.

## Introduction

### Definition

Sleep-disordered breathing (SDB) refers to a wide spectrum of conditions ranging from primary snoring and upper airway resistance syndrome to obstructive sleep apnea (OSA). Primary snoring is deﬁned as snoring without medical comorbidity (1). Upper airway resistance syndrome is characterized by sleep fragmentation with short (1–3 s) arousals preceded by 3–20 s periods of increasing intrathoracic pressure not associated with oxygen desaturation, apnea, or hypopnea (2). OSA is deﬁned by the *American Thoracic Society* as a disorder of breathing during sleep characterized by prolonged partial upper airway obstruction (hypopnea) or intermittent complete obstruction (obstructive apnea) that disrupts normal ventilation during sleep and normal sleep patterns (3).

### Epidemiology

OSA occurs at any age, mostly between 2 and 6 years of age, and affects 2%–4% of children (4, 5). In children, OSA has a lower prevalence and no gender difference compared to adults, ranging from 1.1% in preschool age to 4% in school age (6). Two age peaks in the incidence of OSA in children were described: the first one between 2 and 8 years of age, mostly due to adenotonsillar hypertrophy (ATH), and the second one during adolescence related to weight gain (6).

Based on the underlying pathophysiological mechanism, three clinical phenotypes of pediatric OSA are defined: type I associated with ATH, type II associated mainly with obesity, and type III associated with craniofacial dysmorphism of congenital syndromes (Crouzon syndrome, Arnold–Chiari malformation, Pierre-Robin syndrome, Down syndrome, achondroplasia, etc.) (6). ATH and obesity are the major risk factors for OSA in otherwise healthy children (7). The prevalence of OSA in obese children rises to 60% (8). Conditions that reduce upper airway size (such as craniofacial anomalies) or that affect neural control (such as cerebral palsy) or upper airway collapsibility (such as muscular dystrophy or other neuromuscular disorders) are other risk factors for OSA (9).

Several studies suggested that allergic rhinitis is a risk factor for SDB in children with ATH (10, 11). A systematic review demonstrated a higher prevalence of SDB in children with allergic rhinitis (10). ATH in children with allergic rhinitis could be due to the immunologic response to antigens and other inflammatory stimuli (11). To date, allergic or nonallergic rhinitis is considered a symptom enhancer rather than risk factor for OSA (12).

An increased risk of SDB in childhood was also identified in premature infants, in children with a family history of OSA, and in the African-American ethnicity (13).

### Clinical manifestations

Clinical manifestations of OSA are both nocturnal and daytime symptoms. Although snoring is present in nearly all children with OSA, it is characterized by a low specificity for OSA and cannot reliably distinguish OSA from primary snoring (5). Other nocturnal symptoms include mouth breathing, noisy breathing, pauses in breathing, coughing, or choking in sleep, restless sleep, and nighttime sweating. Nocturnal enuresis and parasomnias such as sleepwalking and sleep terrors are common but less well-recognized symptoms (14).

Daytime sleepiness may manifest as age-inappropriate daytime napping, complaints of sleepiness, or falling asleep during school, short car rides, or on the school bus (15). Mouth breathing or hyponasal speech is common in children with OSA due to its association with adenoidal hypertrophy (16).

Chronic exposure to intermittent hypoxemia and sleep deprivation could lead to neurobehavioral sequelae (5). Behavioral (hyperactivity, impulsivity, rebelliousness, and aggression) and neurocognitive (inattention and learning problems) problems sometimes lead to a misdiagnosis of attention deficit hyperactivity disorder (14).

Severe OSA can be associated with failure to thrive, probably due to the increased energy expenditure for the elevated work of breathing during sleep (17) and the reduced production of growth hormone during disturbed sleep (6).

Furthermore, several studies demonstrated that OSA in children is associated with metabolic, cardiovascular, and neurocognitive complications (15, 18–21). In a 20-year follow-up study of adults with polysomnography (PSG)-documented OSA between 1 and 17 years of age, subjects with severe OSA showed significantly higher BMI, lower academic qualifications, and higher incidence of snoring than a healthy control group. Furthermore, apnea–hypopnea index (AHI) tended to predict cardiovascular outcomes during childhood (21).

At last, symptoms of OSA in children depend on the developmental stage. Disturbed sleep with frequent changes of position and nightmares, growth defects, and behavioral disorders such as hyperactivity and inattention are mostly described in preschool age. Excessive sweating, pavor nocturnus and somnambulism during sleep, hyporexia, learning disabilities, daytime sleepiness, emotional instability, difficulty in morning awakening, bruxism, and diurnal headaches are more common in school age (6).

### Diagnosis

Early diagnosis and treatment of OSA could decrease morbidity. However, diagnosis is frequently delayed (22).

OSA is mostly suspected when specific signs and symptoms were reported by parents or identified by a physical examination (13). History and physical examination are useful for screening subjects who need further investigations but are insufficient to diagnose OSA (6). Measures such as tonsil size assessment, questionnaires, sleep videos, and nocturnal oximetry showed notable variability and inaccuracy in identifying children with OSA (23), and they are mostly used as screening tools in low-resource settings.

PSG is the gold standard for the diagnosis and severity assessment of OSA. PSG is the simultaneous recording of multiple physiological signals during sleep including activity of the brain, heart, eyes, and muscles (24). According to the *American Academy of Otolaryngology—Head and Neck Surgery Foundation*, PSG is indicated in children with SDB and complex medical conditions (obesity, Down syndrome, craniofacial abnormalities, neuromuscular disorders, sickle cell disease, mucopolysaccharidoses) before determining the need for tonsillectomy and in children with SDB and discordance between the tonsillar size and the reported severity of SDB (25, 26).

Sleep and associated events are scored according to the *American Academy of Sleep Medicine Manual* guidelines since 2007 (27), periodically updated (28, 29). Specific guidelines for scoring pediatric sleep were included in the 2007 update (27, 30). During PSG, multiple sensors are used: nasal and oral airflow sensors, snoring microphones, respiratory impedance plethysmographs, pulse oximetry sensors, electrocardiograms, carbon dioxide (CO_2_) sensors, electroencephalography instruments, and body position monitoring systems. Measurement of these variables permits detection of events and calculation of summary measures for the diagnosis and severity assessment of OSA (30–32):

• AHI: the number of apneas plus hypopneas per hour of sleep. AHI > 1 is considered suggestive of OSA in children. AHI between 1 and 4 indicates mild OSA syndrome, AHI between 5 and 9 indicates moderate OSA syndrome, and AHI ≥ 10 indicates severe OSA syndrome;
• Respiratory disturbance index (RDI): the number of apneas, hypopneas, and arousals related to respiratory efforts (RERAs) per hour of sleep; RDI represents the severity parameter; and
• Hypoventilation: end-tidal or transcutaneous CO_2_ > 50 mmHg that persists for more than 25% of the total sleep time.

Given the higher respiratory rate in children compared to adults, the duration of sleep respiratory events was considered differently in defining the pathological respiratory events during sleep (6). While the obstructive event duration in adults must be at least 10 s, the pediatric scoring criteria state that obstructive apneas, hypopneas, and RERA must last ≥2 respiratory cycles (24, 32).

The severity definition differs in children compared to adults: AHI ≥ 5 represents the cutoff for a therapeutic need in children to avoid long-term sequelae; conversely, it is the lower limit value for the definition of disease in adults (6).

At last, awake flexible laryngoscopy, drug-induced sleep endoscopy (DISE), and drug-induced sleep cine magnetic resonance imaging (MRI) are useful diagnostic tools to identify anatomic sites of obstruction in children with OSA. Indeed, findings of endoscopy guide appropriate surgical intervention or identify the level of obstruction in the case of residual OSA after surgical treatment. Similarly, Cine-MRI is a promising tool in children with persistent OSA after AT evaluating the size and volume of the upper airway lumen and surrounding soft tissues (13).

### Treatment

OSA syndrome in children is a heterogeneous condition with different treatment options. The treatment strategy depends on child’s age, underlying etiology, disease severity, PSG findings, comorbidities, and patient beliefs (31, 33).

Surgical treatment with adenoidectomy, tonsillectomy, or AT is the first-line treatment for children with moderate-to-severe OSA syndrome (AHI ≥ 5) aged >2 years old and enlarged adenoids or tonsils (6, 34). It is a safe procedure, with 93% of patients without perioperative complications and a success rate of 75%. Postoperative PSG typically shows a major decrease in obstructive events (23).

Adenoidectomy or tonsillectomy alone may not be sufficient because residual lymphoid tissue may contribute to persistent obstruction (23).

In addition to the risks related to anesthesia, the most common complications include nausea, vomiting, postoperative pain, and bleeding. Less common complications are dehydration, referred otalgia, postobstructive pulmonary edema, velopharyngeal insufficiency, and nasopharyngeal stenosis (35).

Residual OSA after AT is common. Recently, Alsufyani et al. (36) investigated predictors of AT failure in 382 children with SDB. The authors identified chronic rhinitis, obesity, deviated nasal septum, small tonsil size, and age older than 7 years as independent predictors of treatment failure.

A recent retrospective study of 139 children with OSA investigated the less effectiveness of AT in obese children compared to normal-weight ones and found an association between body mass index and circumferential upper airway collapse during DISE. The authors showed that continuous positive airway pressure (CPAP) is more effective compared to AT in children with circumferential collapse (37).

Adenoidectomy alone could be considered, especially in children younger than 2 years of age, to avoid life-threatening hemorrhage in young patients (38).

CPAP is considered a second-line therapy for residual OSA post-AT or bridging therapy before surgery. It is considered a first-line therapeutic option for children who prefer not to undergo surgery, those with minimally enlarged lymph adenoid tissues not indicated for AT, or those with comorbidities with multilevel obstruction, such as obesity and craniofacial syndromes (33).

Medical options could be the best choice for children with an AHI ≥ 1 but <5. Based on the evidence of inflammation and the coexistence of rhinitis and asthma in OSA, anti-inflammatory medications, mainly intranasal corticosteroids and oral montelukast, were used as adjunctive treatments (6, 39).

At last, dental/orthodontic treatment options have emerged in the past decade for children with OSA and orofacial abnormalities. These include rapid maxillary expansion, mandibular advancement appliance, or maxillo-mandibular surgery (40).

A re-evaluation 6–8 weeks after treatment and instrumental tests in patients with residual symptoms are recommended (41).

## Case description

A Caucasian 4-year-old boy was admitted to the Pediatric Department for parent-reported episodes of sleep apnea in the last 3 months. Nocturnal symptoms were snoring, mouth breathing, and excessive sweating; daytime symptoms were asthenia, daytime sleepiness, inattention, and reduced school performance.

The child suffered from recurrent upper airway infections and chronic obstructive rhinitis since the age of 18 months. For this reason, some investigations were performed on the child between 2.5 and 3 years of age. The immunological evaluation by cell blood count and serum immunoglobulin and lymphocyte subpopulation dosage resulted in normal; the allergy evaluation by a skin prick test and the dosage of total and specific immunoglobulin E for the main food and respiratory allergens resulted in normal; the ear, nose, and throat (ENT) evaluation found severe tonsillar hypertrophy and indicated an adenotonsillectomy (AT). While waiting for surgery, the child underwent unsuccessful therapy with nasal steroids.

Additionally, the child began to present a weight gain from 3 years of age. The parents reported that the weight gain began with the increasing use of oral cortisone. At 4 years of age, the child was obese, with a body weight of 25 kg and a height of 116 cm, resulting in a body mass index of 18.6 kg/m^2^ (98th percentile).

Polygraphy (PG) was performed 2 weeks before admission. The study was recorded on a *Philips Respironics Alice PDx* device with pediatric sensors.

PG lasted 477 min and documented 24 obstructive apneas, 14 central apneas, 39 hypopneas, 9 mixed apneas, and no RERA. The mean oxygen saturation values resulted in 98%, with a minimum of 72%. The percentage of sleep time spent with oxyhemoglobin saturation of less than 90% was 0.6%. The mean heart rate was 83.7 beats per minute (bpm). The patient frequently changed position during monitoring. Flow limitations were detected in all the positions during sleep analysis. Therefore, PG documented severe OSA syndrome with an AHI equal to 11, 3.1 episodes of obstructive apnea, and 1.8 episodes of central apnea in 1 h.

**Figure 1:**
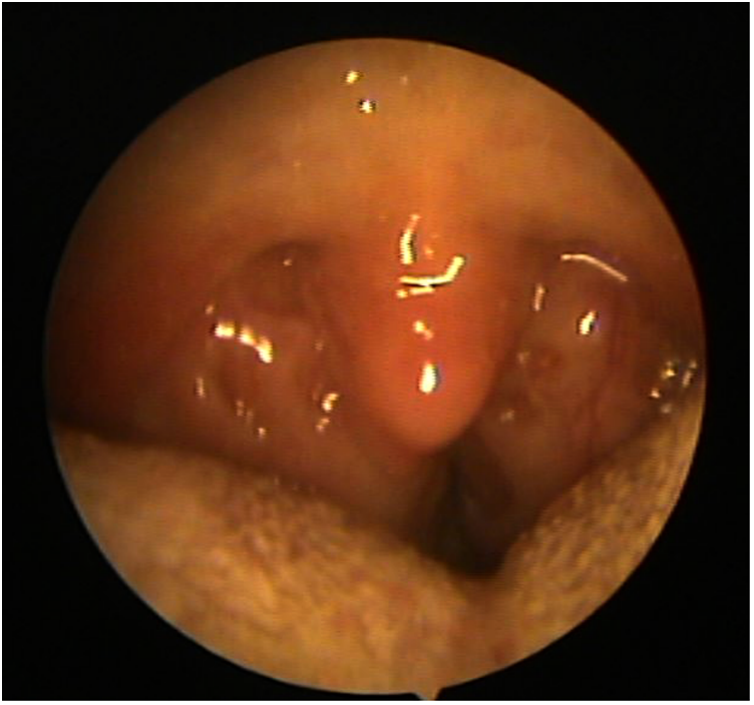
Ear, nose, and throat specialist found severe tonsillar hypertrophy (*kissing tonsils*).

After PG, a second ENT consultation documented “no polyps in the nasal cavities or deviation of the nasal septum; mild adenoid hypertrophy; tonsillar gigantism; no anatomical anomalies of the oral cavity and no post-nasal drip; opaque tympanic membranes” (Figure 1) and indicated a prompt surgical removal of the tonsils to facilitate airflow through the airways. A dissection tonsillectomy in the Rose position (open technique) was performed with no following complications.

PG was performed 3 weeks after tonsillectomy; the test lasted 509 min and documented 1 obstructive apnea, 3 central apneas, 12 hypopneas, 3 mixed apneas, and no RERA. The mean oxygen saturation values was 97%, with a minimum of 93%. The mean heart rate was 72.3 bpm. PG showed a substantial reduction of AHI (2.2). The PG report before and after the surgical treatment is shown in Table 1.

**Table 1:** Polygraphy report before and after the surgical treatment. Polygraphy before surgery lasted 477 min and documented 24 obstructive apneas, 14 central apneas, 39 hypopneas, 9 mixed apneas, and no arousals related to respiratory effort (RERA). The mean oxygen saturation values was 98%, with a minimum of 72%. The percentage of sleep time spent with oxyhemoglobin saturation of less than 90% was 0.6%. Polygraphy (PG) documented severe obstructive sleep apnea syndrome with an apnea–hypopnea index (AHI) equal to 11, 3.1 episodes of obstructive apnea, 1.8 episodes of central apnea in 1 h, and the lowest desaturation value of 72%. Three weeks after surgery, PG lasted 509 min and documented 1 obstructive apnea, 3 central apneas, 12 hypopneas, 3 mixed apneas, and no RERA. The mean oxygen saturation values was 97%, with a minimum of 93%. PG showed a substantial reduction of AHI (2.2).

	Before tonsillectomy	After tonsillectomy
*Respiratory events*		
Central apnea (*n*)	14	3
Obstructive apnea (*n*)	24	1
Mixed apnea (*n*)	9	3
Hypopnea (*n*)	39	12
Apnea + hypopnea (*n*)	86	19
RERA (*n*)	0	0
Total (*n*)	86	19
*Oximetry summary*		
Total sleep time SpO_2 _< 90% (min)	2.8	0.0
Mean SpO_2_ (%)	98	97
Total desaturations (*n*)	68	22
Oxygen desaturation index (*n*/h)	12.9	2.6
Desaturation max (%)	6	5
Duration max desaturation (s)	56	44
Lowest sleep SpO_2_ (%)	72	93
Duration lowest SpO_2_ (s)	83	2
Maximum SpO_2_ during sleep (%)	100	99
Duration maximum SpO_2_ (s)	569	45
*Mean heart rate (bpm)*	83.7	72.3
*Snoring summary*		
Snoring episodes (*n*)	5	23
Total duration with snoring (min)	0.6	2.6
Mean snoring duration (s)	6.8	6.8
Percentage of snoring (%)	0.1	0.5
*Apnea–hypopnea index (AHI)*	11.0	2.2
*Obstructive apnea for hour (OAI)*	3.1	0.1
*Central apnea for hour (CAI)*	1.8	0.4

At the follow-up visit 1 month after the surgical procedure, the parents reported complete disappearance of nocturnal symptoms and an improvement in daytime sleepiness. Four months after surgery, the parents reported a remarkable improvement in the quality of life, thanks to the disappearance of nocturnal symptoms and less daytime sleepiness. Unfortunately, the child was still obese and had recurrent respiratory infections. A further allergy and immunological function evaluation showed only a slight increase in dust mite-specific immunoglobulin E (0.55 kUA/L).

**Figure 2:**
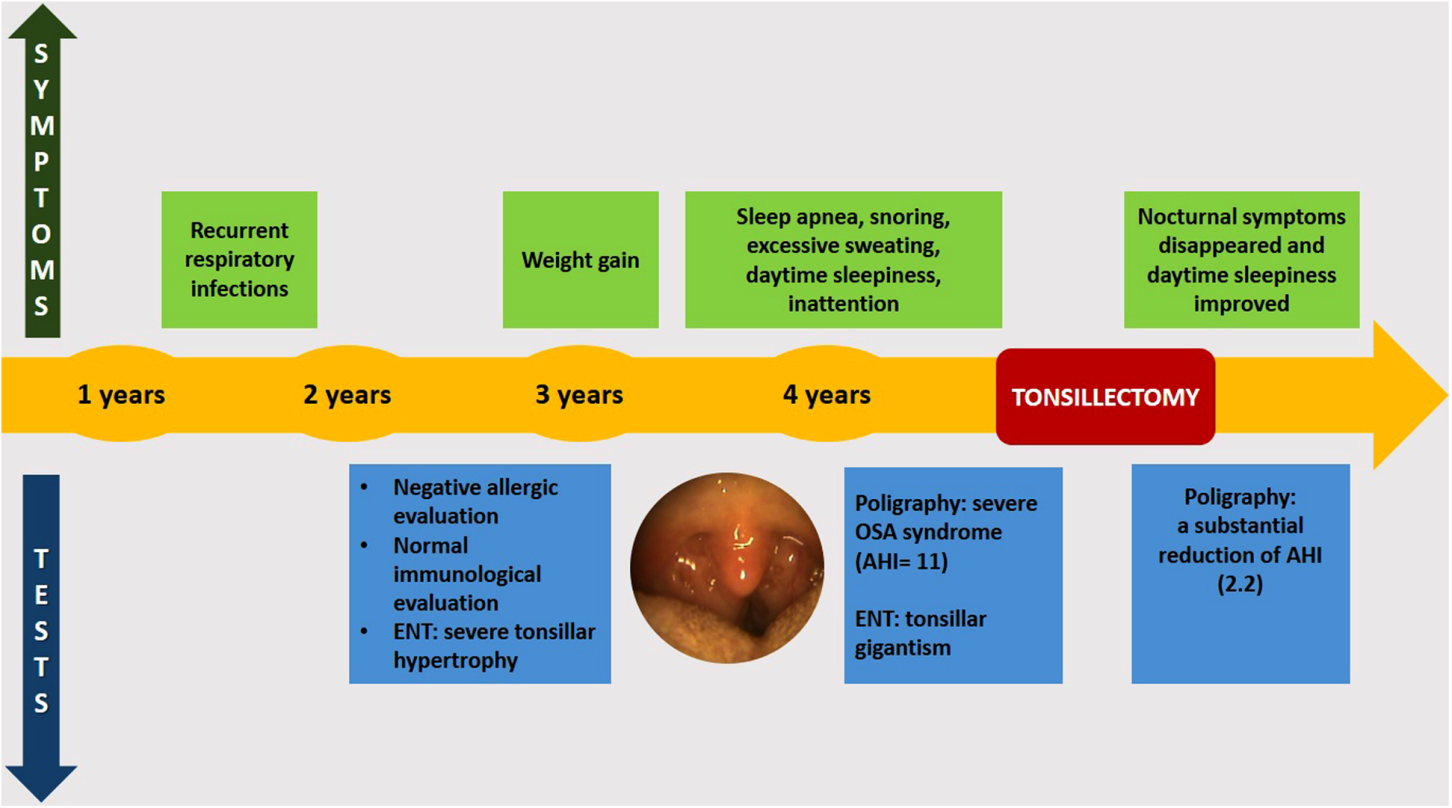
Timeline of the clinical course before and after surgery. ENT, ear, nose, and throat; OSA, obstructive sleep apnea; AHI, apnea–hypopnea index.

Figure 2 shows the timeline of the clinical course of the child before and after surgery.

Written informed consent was obtained from the minor’s legal guardian.

## Discussion

Symptoms of OSA in our patient started at 4 years of age, in line with the literature; indeed, OSA in children occurs mostly between 2 and 6 years of age (4, 5). The child's parents complained of snoring, which is the most common symptom of OSA (5). The parents also reported pauses in breathing but no other nocturnal symptoms, although these were common but less recognized (14).

The child also suffered from daytime sleepiness, usually more common in school age (6), while daytime symptoms of OSA typical of preschool age (as behavioral disorders) were not reported.

The child also complained of chronic nasal obstruction. Rhinitis often coexists with OSA, and patients with OSA report nasal obstruction in 54% of cases (42, 43). Our patient was not allergic, but a nasal provocation test or nasal cytology to rule out nonallergic rhinitis was not performed. Nevertheless, in our patient, local steroid therapy did not improve symptoms.

In our case, the child presented both major risk factors for OSA: ATH and obesity (7). In children, the type I clinical phenotype of OSA is associated with ATH and occurs mostly between 2 and 8 years of age and the type II phenotype is associated mainly with obesity and occurs mostly in adolescence age (6). Therefore, our patient presented a bridging phenotype between type I and type II.

Although the evidence showed that obesity is a major cause of sleep disorders, it was recently speculated that sleep disorders might cause obesity. In the literature, higher circulating levels of leptin and thus leptin resistance were found in obese patients with OSA compared to obese patients without OSA (44, 45). Hyperleptinemia could originate in response to intermittent hypoxia (46), stimulating food intake in subjects with poor sleep and resulting in a higher risk of developing obesity (45). Conversely, other studies observed no alterations in leptin levels in OSA patients, hypothesizing that increased levels of leptin originated from obesity rather than sleep disorders (47, 48). To date, the causal relationship between obesity and sleep disorders is still unclear. In our patient, it was not possible to establish whether obesity caused OSA or vice versa, as the interval between ATH and obesity and OSA was too short. However, good response to tonsillectomy suggested that ATH was the determinant cause and obesity was an aggravating factor of OSA.

A prompt diagnosis of OSA is important to address an appropriate treatment and avoid complications. In our case, nasal steroid treatment failed, and the clinical evaluation showed tonsillar gigantism and PSG-documented severe OSA syndrome. Therefore, ENT consultation indicated a prompt surgical removal of the tonsils. AT is the first-line treatment for children aged >2 years old with moderate-to-severe OSA and enlarged adenoids or tonsils (6, 33). The child presented chronic rhinitis and obesity, which are predictors of AT failure. Persistent OSA after AT was found in 33%–76% of obese children compared to 15%–37% of nonobese children (49). Therefore, greater efficacy of CPAP compared to AT in obese patients with OSA was suggested (33, 37).

Many studies hypothesized that the patient's age might influence the success rate of AT in obese children (49, 50). The authors suggested that obesity acts as an OSA risk enhancer in younger children, in whom ATH is the main cause of OSA and emerges as a major OSA determinant in older children. In addition, 464 children with OSA were randomly assigned to early AT or a strategy of watchful waiting in a randomized controlled trial. The authors found a significant improvement in symptoms and behavioral, quality-of-life, and polysomnographic findings in the early AT group than in the watchful-waiting group, demonstrating the beneficial effects of early AT (51). The young age of our patient and the presence of ATH were considered more than obesity for the therapeutic choice.

Three weeks after tonsillectomy, PG documented a substantial reduction of AHI (2.2), and a complete regression of symptoms was reported. Surgery success was defined as a postoperative reduction of AHI to <20 and AHI > 50% (52, 53). In our case, the reduction of AHI from 11 to 2.2 showed a positive outcome of the surgery treatment. Tonsillectomy without adenoidectomy produced sufficient results in the child, although a persistent obstruction due to residual lymphoid tissue was reported in the literature (23).

However, a longer follow-up would be needed to identify the long-term effects of surgical therapy.

## Conclusion

OSA is a common disorder in children with negative consequences, potentially detrimental to long-term health. An early diagnosis and prompt treatment can prevent cardiovascular, metabolic, and neurocognitive consequences and improve long-term cognitive performance. Therefore, it is necessary to identify, investigate, and promptly treat children with suggestive symptoms and those at risk for OSA syndrome. However, diagnosis and management of OSA syndrome in children are not easy, especially because of the still poor knowledge of the underlying pathogenic mechanisms and the factors influencing the phenotypic variability.

ATH is a predictor of severe OSA, and children with this condition should be prioritized for early PSG and management.

Obesity is another important risk factor for OSA, and the growing number of obese children worldwide is a cause of concern when managing children with OSA. In addition, obesity is a predictor of AT failure. However, the presence of obesity should not postpone/exclude surgical treatment of preschool children for whom ATH is the most important cause of OSA.

## Data Availability

The original contributions presented in the study are included in the article/Supplementary Material; further inquiries can be directed to the corresponding author.
